# Turning Complexity into Simplicity: In Situ Synthesis of High-Performance Si@C Anode in Battery Manufacturing Process by Partially Carbonizing the Slurry of Si Nanoparticles and Dual Polymers

**DOI:** 10.3390/molecules29010175

**Published:** 2023-12-28

**Authors:** Xiaoxian Liu, Juan Liu, Xiaoyu Zhao, Dianhong Chai, Nengwen Ding, Qian Zhang, Xiaocheng Li

**Affiliations:** 1 Jiangxi Province Key Laboratory of Power Battery and Materials, Faculty of Materials Metallurgy and Chemistry, Jiangxi University of Science and Technology, Ganzhou 341000, China; aa13184544928@163.com (X.L.); caiweibest@126.com (X.Z.); cdh6662021@126.com (D.C.); ding_0321@126.com (N.D.); zhangqian@jxust.edu.cn (Q.Z.); 2 Jiangxi Province Key Laboratory of Mining Engineering, School of Resources and Environmental Engineering, Jiangxi University of Science and Technology, Ganzhou 341000, China; 3 Yichun Lithium New Energy Industry Research Institute, Jiangxi University of Science and Technology, Yichun 360904, China

**Keywords:** silicon anode, partial carbonization, dual polymer, in situ synthesis, dual-interfacial bonding

## Abstract

For Si/C anodes, achieving excellent performance with a simple fabrication process is still an ongoing challenge. Herein, we report a green, facile and scalable approach for the in situ synthesis of Si@C anodes during the electrode manufacturing process by partially carbonizing Si nanoparticles (Si NPs) and dual polymers at a relatively low temperature. Due to the proper mass ratio of the two polymer precursors and proper carbonization temperature, the resultant Si-based anode demonstrates a typical Si@C core–shell structure and has strong mechanical properties with the aid of dual-interfacial bonding between the Si NPs core and carbon shell layer, as well as between the C matrix and the underlying Cu foil. Consequently, the resultant Si@C anode shows a high specific capacity (3458.1 mAh g^−1^ at 0.2 A g^−1^), good rate capability (1039 mAh g^−1^ at 4 A g^−1^) and excellent cyclability (77.94% of capacity retention at a high current density of 1 A g^−1^ after 200 cycles). More importantly, the synthesis of the Si@C anode is integrated in situ into the electrode manufacturing process and, thus, significantly decreases the cost of the lithium-ion battery but without sacrificing the electrochemical performance of the Si@C anode. Our results provide a new strategy for designing next-generation, high-capacity and cost-effective batteries.

## 1. Introduction

In recent years, the explosive growth of electronic devices and electrical vehicles has led to an exigent requirement for high-performance energy storage systems (ESSs) [1,2]. Among all EESs, lithium-ion batteries (LIBs) have a dominant market share because of their high energy density, high operating voltages, good compatibility and long lifespan [3]. Despite remarkable progress, LIBs are still required to have a higher energy density for a wide market requirement, especially in the field of electrical vehicles. Currently, research on improving the specific capacity of cathode materials for LIBs has reached a bottleneck [4], and the specific capacity of some cathode materials is approaching their theoretic capacity, for example, LiFePO_4_ [5]. Under this circumstance, replacing the commercial graphite (372 mAh g^−1^, LiC_6_) anode with a new anode material is an alternative strategy to further improve the energy density of LIB devices. Silicon (Si), with an extremely high theoretical capacity (3579 mAh g^−1^, Li_15_Si_4_), relatively low lithiation potential (<0.5 V) and abundant earth reserves, has been considered one of the most promising anode materials for next-generation LIBs. However, the significant volume expansion of Si (~300%) during the lithiation/delithiation process results in serious problems for the performance of electrodes, such as extremely low initial Coulombic efficiency (ICE), continuous growth of the solid electrolyte interphase (SEI) layer and fast capacity fading. Countless approaches have been proposed to mitigate the volume expansion of Si-based electrodes by adopting the following strategies: (1) decreasing the size of Si materials or lowering the dimension of Si materials [6,7]; (2) developing diverse coating routes to consolidate the structure of Si species [8,9]; (3) constructing void-containing Si/C or Si/TiO_2_ composites [10,11]; (4) constructing a Si-based micro/nano binary structure [12]; and (5) adding chemical additives to the electrolyte to stabilize the SEI layer [13]. The proposed approaches effectively buffer the volume expansion of Si and significantly improve the electrochemical performance of Si anodes. However, most of these approaches involve either tedious multistage synthesis procedures or expensive equipment. Therefore, the fabrication of high-performance Si-based anodes using a facile approach is still an ongoing challenge. But almost certainly, based on previous reports, carbon coating is an effective strategy to stabilize the structure of Si and can improve the electrical conductivity of the Si-based anodes.

In addition to the structure of the Si host materials, the manufacturing process is also an important factor in determining the cost and electrochemical performance of the Si-based electrodes [14]. Optimizing the manufacturing process can not only effectively reduce the cost of electrodes but can also significantly improve their electrochemical performance, such as capacity, cyclability, cost and safety [15]. Currently, negative electrodes are manufactured by coating the slurry consisting of active material (for example, Si/C composite), polar polymer binder (for example, polyacrylonitrile [16], polyimide [17] and polyvinyl alcohol [18]) and conductive agents followed by drying and roll pressing. For Si-based electrodes, the mass fraction of the binder and conductive agents is high and can reach up to 40 wt% [19], which sharply abates the electrochemical performance of Si anodes, especially their specific capacity. Moreover, current reports on the electrochemical performance of Si anodes are based on the mass of the electroactive Si/C composite without considering the mass of the binder and conductive agents. Therefore, if the synthesis procedure of the Si/C composite can be integrated into the manufacturing process of electrode plates (namely, the in situ synthesis of Si/C composites during the manufacturing process of negative electrode plates), high performance of Si-containing negative electrodes can be expected.

With these in mind, in this study, we propose a green, facile and versatile approach to the in situ synthesis of the Si@C-based anode during the electrode manufacturing process by directly carbolizing a slurry mixture of commercial Si NPs, polyacrylic acid (PAA) and poly(cyclotriphosphazene-co-(4,4′-sulfonyldiphenol)) (abbreviated as PZS) on Cu foil at relatively low temperatures. As a commonly used binder in Si-based anodes, PAA is rich in carboxyl groups and easily interacts with silanol groups (Si-OH) on Si NPs or Cu-O bonds on the native oxide layer of Cu foil via hydrogen bonds [20], which is attributed to the dispersion of Si NPs and enables the formation of a uniform PAA–Si NP film on the Cu foil. The carbon film resulting from the carbonization of PAA (denoted as C_A_) can significantly improve the electric conductivity of the Si anode. To overcome the drawbacks of PAA precursors, such as low carbon content and emission of CO_2_ during the carbonization process, another polymer, PZS, was chosen as a carbon source due to its good wettability, long-term colloidal stability and good water dispersibility [21], and the corresponding carbon film was denoted as C_z_. The heteroatoms (N and P) in PZS molecules also prompt their dispersibility because of the strong hydrogen bonds between the heteroatoms in PZS and the carboxyl groups in PAA molecules. The heteroatoms in PZS molecules also can further improve the conductivity of the carbonized PAA–PZS polymers (denoted as C_AZ_), as well as that of Si@C_AZ_ composite. Moreover, the carbonized PAA–PZS forms a 3D pathway for the transportation of Li^+^/electron without adding extra wildly used conductive carbon agent (for example, Super P) and, thus, would significantly improve the capacity of the LIB device. Thanks to these advantages, the as-prepared Si@C_AZ_-based electrode demonstrates high specific capacity, good rate capability and excellent cyclability. More importantly, we proposed a facile and versatile strategy which integrates all processes, including carbonization of PAA–PZS dual polymers, preparation of Si@C_AZ_ core–shell structure and electrode manufacture process, into one step, thus dramatically decreasing the cost of the LIBs and simultaneously achieving excellent electrochemical performance, suggesting its promising applications in LIBs.

## 2. Results and Discussion

### 2.1. Structural Characterization

**Figure 1:**
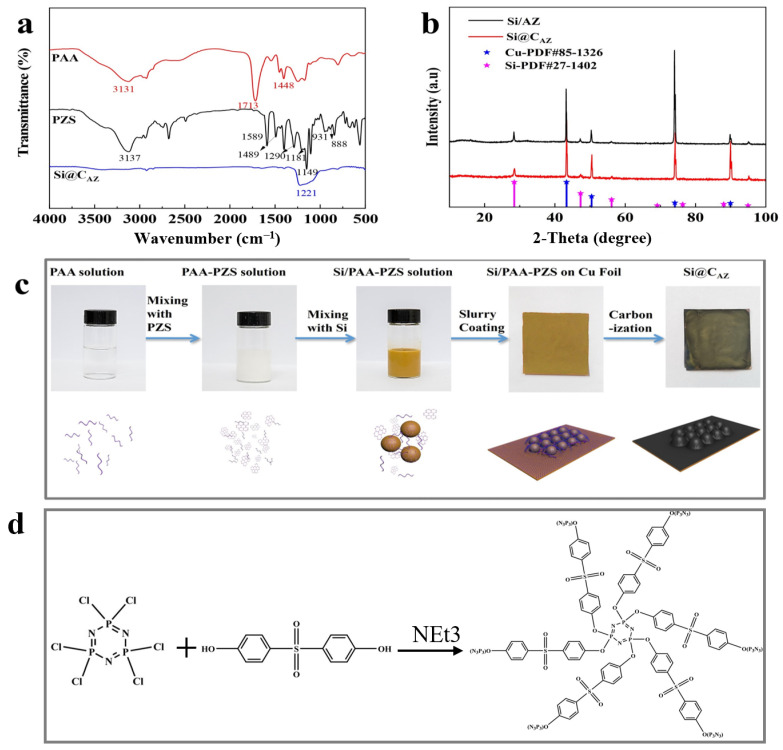
(**a**) FT-IR spectra of PAA, PZS and Si@C_AZ_; (**b**) XRD patterns of Si/C_AZ_ and Si@C_AZ_; (**c**) schematic diagram of the procedure for preparing Si@C_AZ_/Cu electrode; and (**d**) formula of the synthesized PZS.

**Figure 2:**
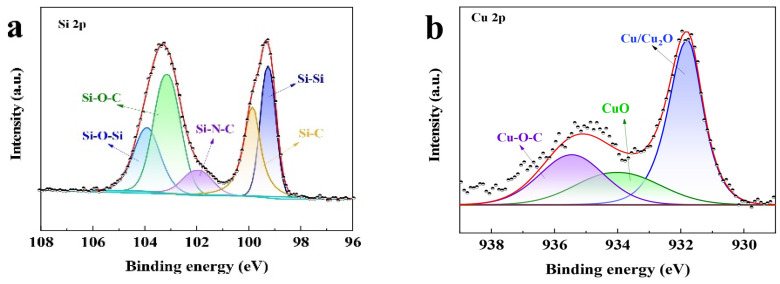
Core-level XPS spectra of (**a**) Si 2p and (**b**) Cu 2p in Si@C_AZ_/Cu electrode.

In this study, the proposed Si@C_AZ_ anode is made through partial carbonization of the slurry of commercial Si NPs and PAA- PZS dual polymers on Cu foil. The PZS is synthesized through a condensation polymerization reaction, and the resultant product was characterized by using an FTIR spectrometer. As shown in Figure 1a, the stretching vibration of C=C (1589, 1489 cm^−1^), O=S=O (1290, 1149 cm^−1^), P=N (1181 cm^−1^) and P-N (888 cm^−1^) bonds can be clearly detected [22]. The stretching vibration at 931 cm^−1^ indicates the presence of P-O-(Ph) bond. The peak at 3137 cm^−1^ belongs to the -OH bond in physically adsorbed water. All these peaks suggest the successful synthesis of the PZS precursor [23] and the structure of the synthesized PZS is depicted in Figure 1d. As another precursor component of Si@C_AZ_, PAA is also characterized by using FTIR. Also, as shown in Figure 1a, the strong adsorption band at 1713 cm^−1^ can be assigned to the stretching vibration of the C=O group in PAA molecules. The two weak peaks at 2875 cm^−1^ and 1448 cm^−1^ can be attributed to the stretching vibration and asymmetric formation vibration of the -CH_2_ group in PAA molecules, respectively. The strong peak at 3113 cm^−1^ can be ascribed to the stretching vibration of OH in -COOH group. The strong -OH bond allows PAA to dissolve in water and mixes with PZS. The strong hydrogen bond between the -OH group in PAA molecule and the Si-O bond on Si NP surface can boost the dispersion of Si NPs in water (Figure 1c). After carbonizing at 450 °C in Ar atmosphere, the resulting composite was used for further structural characterization and electrochemical performance evaluation. From the optical image, as shown in the right two panels of Figure 1c, the color of the sample changes from brown–yellow to dark green, indicating the formation of the carbon species during the carbonization process. XRD patterns in Figure 1b show that the Si@C_AZ_ only contains Si and carbon resulting from the carbonization of PAA–PZS dual polymers, and no undesirable by-products are formed during the pyrolysis process. FTIR spectra of Si@C_AZ_ (Figure 1a) demonstrate that, after carbonization, most oxygen-containing groups disappear. For PAA, the stretching vibration of C=O is faintly visible and the in-plane vibration of the δ_C-H_ can be clearly detected. For PZS, only some polar groups within 1500–1000 cm^−1^ and some carbon-containing groups (C=C) at 1489 and 1589 cm^−1^ can be detected. The broad absorption peaks within 1500–1000 cm^−1^ are closely related with the Si-C or Si-O-C bond, which will be verified by XPS spectra. The XPS survey spectrum indicates the existence of Si 2p, C 1s, N 1s and O 1s signals in Si@C_AZ_ (Figures S1 and S2). In Si 2p spectra (Figure 2a), the characteristic peaks at 99.4 and 103.9 eV correspond to Si-Si and Si-O-Si bond, respectively. In addition, another three peaks centered at 103.2, 102.0 and 99.8 eV also can be observed, which can be assigned to the Si-O-C, Si-N-C and Si-C bonds, respectively [24,25], suggesting the strong chemical bond between Si NPs and C_AZ_ matrix, as well as between Si NPs and heteroatoms in C_AZ_. The formation of Si-O-C, Si-C and Si-N bonds can effectively enhance the interaction between the C_AZ_ and the Si NPs. In Cu 2p3/2 XPS spectra (Figure 2b), the strong Cu-O-C bond (935.4 eV) also can be clearly detected [26], indicating the strong chemical interaction between the C_AZ_ and the native oxide layer on Cu foil. Due to the strong Cu-O-C bond, the Si@C_AZ_ particles can be firmly anchored on Cu foil, and no cracks or delamination of the Si@C_AZ_ from Cu foil can be observed, even after folding several times (Figure S3), indicating the excellent mechanical flexibility and stability of the as-prepared Si@C_AZ_/Cu electrode. Obviously, the dual-interfacial bonding (Si-O-C, Si-C, Si-N and Cu-O-C bonds) formed during the pyrolysis process endows the Si@C_AZ_/Cu electrode with good mechanical strength via the interaction between C_AZ_ and Si NPs, as well as between Si@C_AZ_ NPs and the underlying Cu foil [27]. In addition, the cross-linked C_AZ_ matrix can act as a conductive framework, which increases the conductivity of the Si@C_AZ_/Cu electrode [28]. All these features make Si@C_AZ_/Cu a robust electrode with strong mechanical properties and good flexibility.

**Figure 3:**
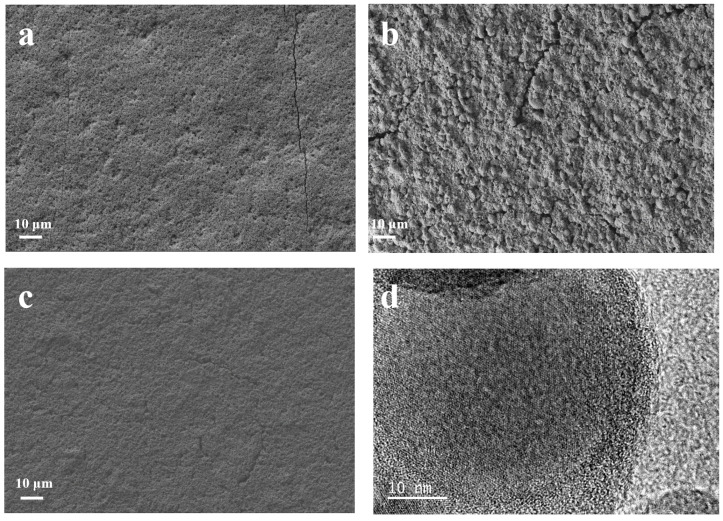
FESEM images of (**a**) Si@C_A_, (**b**) Si@C_Z_ and (**c**) Si@C_AZ_, and (**d**) HRTEM image of Si@C_AZ_ composite.

Scanning electron microscopy is used to observe the surface morphology of the as-prepared samples. As shown in Figure 3a, the as-prepared Si@C_A_ electrode shows a smooth surface due to the good flowability of the PAA. Additionally, few cracks and large amounts of pits resulting from the emission of CO_2_ and H_2_O during the carbonization of the PAA molecules are also observed. For Si@C_Z_-based electrode, due to the weak interaction between PZS molecules, an uneven and rough surface with rock-like morphology is observed (Figure 3b). In addition, some non-negligible cracks are also observed. When PAA–PZS dual polymers are used as a carbon source, the prepared Si@C_AZ_ electrode shows a smooth surface, and no cracks or pits can be observed (Figure 3c). HRTEM image of the Si@C_AZ_, as shown in Figure 3d, shows that Si NPs are wrapped by the C_AZ_ film with a thickness of ~4 nm. The TGA analysis result indicates that the carbon content in Si@C_AZ_ composite is only 3.89 wt% (Figure S4). Despite the extremely low carbon content, from FESEM and HRTEM images, the uniform coating of the carbon layer on Si NPs can still be guaranteed and no bare Si NPs or Si NP aggregations can be observed. The uniform C_AZ_ coating on the Si NP surface, together with the C_AZ_ matrix in Si@C_AZ_ composite, provides a continuous pathway for the transportation of the lithium ions and electrons during the lithiation/delithiation process. Since the C_AZ_ fraction in Si@C_AZ_ composite is extremely low, the addition of the C_AZ_ will not remarkably blemish the capacity of Si@C_AZ_ composite but will significantly improve the rate capacity and cyclability of the Si@C_AZ_ composite.

The effects of carbonization temperature and PAA/PZS ratio on the morphology of the Si@C_AZ_ samples were also systematically investigated. As shown in Figure S5a, the Si@C_AZ_ annealed at 350 °C shows a relatively smooth surface with few cracks, indicating the effectiveness of polar groups in PAA at low temperature. When the annealing temperature is increased up to 550 °C, the resulting Si@C_AZ_-550 (Figure S5b) electrode is dominated by serious cracking. This implies that too high a carbonization temperature (550 °C) would destroy the polar groups in PAA molecule and make it lose the role of bonding. Therefore, 450 °C is a suitable carbonization temperature under which the PAA–PZS is partially carbonized and cracks are thereby avoided. In addition, the effect of PAA/PZS ratio on the morphology of the as-prepared sample is also explored. The Si@C_Z_ electrode, by carbonizing the Si NPs and PZS, demonstrates an uneven and rough surface with rock-like morphology. When the PAA/PZS is changed as 25:75 followed by annealing at 450 °C, the surface of the as-prepared Si@C_A25Z75_ electrode is flatter but some pits still can be observed (Figure S5c). When the PAA/PZS ratio is 50:50, the Si@C_AZ_ electrode shows a smooth surface and no cracks or pits can be observed (Figure 3c). For Si@C_A75Z25_ electrode, from the non-negligible big fissure that formed on the sample surface, it can be observed that the Si@C_A75Z25_ sample consists of two layers, namely, a smooth upper layer resulting from partial carbonization of PAA molecules and a rough bottom layer originating from the carbonization of PZS molecules (Figure S5d). Although Si NPs are mobilized within both carbon layers, the obvious carbon separation in Si@C_A75Z25_ sample would have a detrimental effect on its electrochemical performance. As the polymer completely consists of the PAA, the as-prepared Si@C_A_ demonstrates a smooth surface but with large amounts of pits and few long cracks due to the fragility of the PAA-derived carbon (Figure 3a). Therefore, based on the surface morphologies of Si@C_AZ_ with different carbonization temperatures and different PAA/PZS ratios, it can be preliminarily determined that the optimal carbonization temperature is 450 °C and the optimal proportion of the PAA binder in dual polymer is 50%, namely, the optimal PAA/PZS ratio is 1:1. All these deductions will be verified by their electrochemical performance in the following section.

### 2.2. Electrochemical Performance Evaluation

**Figure 4:**
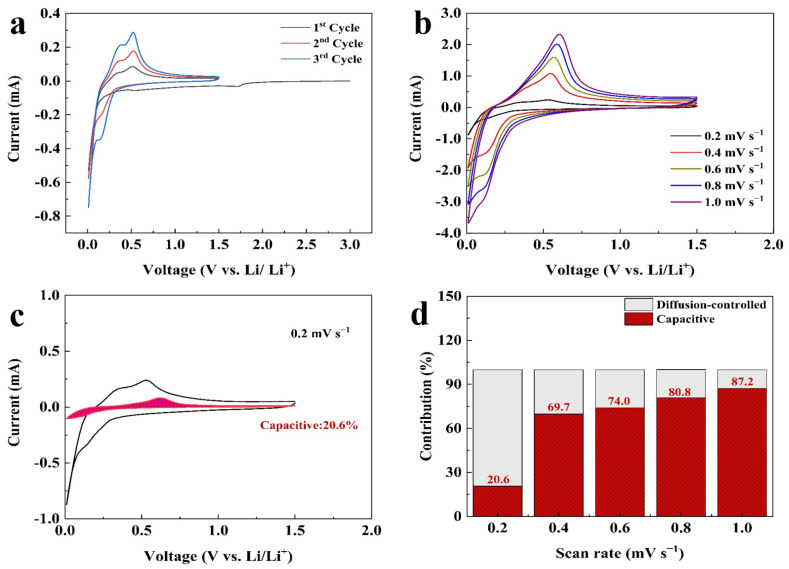
Electrochemical performance of the Si@C_AZ_ electrode: (**a**) CV curves at 0.1 mV s^−1^, (**b**) CV curves at scan rates of 0.2–1.0 mV s^−1^, (**c**) separation of capacitance and diffusion-controlled process at 0.2 mV s^−1^, (**d**) capacity contributions at scan rates of 0.2–1.0 mV s^−1^.

The electrochemical performances of all prepared electrodes were evaluated within the potential range of 1.5–0.01 V. Figure 4a shows the initial three CV curves of the Si@C_AZ_ anode at a sweep rate of 0.1 mV s^−1^. In the cathodic branch of the first cycle, the reduction peak at ~1.72 V is related to the reduction of FEC additive in electrolyte, which enables the uniform growth of SEI film on Si@C_AZ_ anode and, thus, improves the cycling stability of the electrode. The sharp peak below 0.05 V represents the alloy reaction of Li^+^ with crystallized Si NPs and involves several Si-containing phases, such as Li_12_Si_7_, Li_14_Si_6_, Li_13_Si_4_ and Li_15_Si_4_ phases, via the reaction Si (crystalline) + xLi^+^ + xe^−^ → LixSi (amorphous) + (3.75 − x)Li^+^ + (3.75 − x) e^−^ → Li_15_Si_4_ (crystalline) [29]. In the subsequent cycles, this peak evolves into the reduction peak at 0.152 V, representing the insertion process of lithium in amorphous Si [30,31]. Compared with that of the pristine Si, the reduction peak at 0.152 V in Si@C_AZ_ composite slightly shifts down to lower potential, which may be attributed to the influence of the carbon layer originating from the carbonization of PAA–PZS dual polymers [32]. In the anodic branch, two obvious peaks at 0.375 V and 0.51 V can be clearly observed, which can be attributed to the dealloying reaction of Li_x_Si alloy and the formation of amorphous Si and involves the reaction Li_15_Si_4_ (crystalline) → Si (amorphous) + yLi^+^ + e^−^ [33]. To determine the contribution of capacitive and diffusion-controlled process in the electrochemical performance of Si@C_AZ_ at different current densities, the as-prepared Si@C_AZ_ electrode was subjected to CV measurement at different sweep rates ranging from 0.2 mV s^−1^ to 1.0 mV s^−1^ (Figure 4b). It is revealed that the oxidation peak slightly moves to the higher potential with the increase in scan rate, while the reduction peak slightly moves to lower potential. Moreover, with the increase in the sweep rate, the broad anodic peak at ~0.55 V gradually envelops the peak at ~0.35 V due to the large current density and high *I-V* response of the delithiation process at ~0.55 V. Despite the slight shift of the redox peaks and the broadness of the peaks at ~0.55 V, the CV curves at high sweep rates still reserve their shapes as those at the low sweep rate (Figure 4a). Based on the CV curves of the Si@C_AZ_ electrode at various sweep rates, capacitive capacity and the capacity resulting from the diffusion-controlled process were calculated by using the method established in the previous report [34]. As shown in Figure 4c, at the low scan rate of 0.2 mV s^−1^, the capacitive capacity accounts for ~20.6% of the total charge storage, indicating the dominant role of the diffusion-controlled process at low sweep rate or low current density. With the increase in scan rate, the contribution of the capacitive capacity remarkably increases and reaches 87.2% at a high sweep rate of 1 mV s^−1^, implying the dominant role of pseudocapacitive process at high scan rate or high current density (Figure 4d). The increased pseudo-capacitance contribution at high sweep rate may result from the core–shell structure of the Si@C_AZ_ in accumulating residual Li ions [35,36].

**Figure 5:**
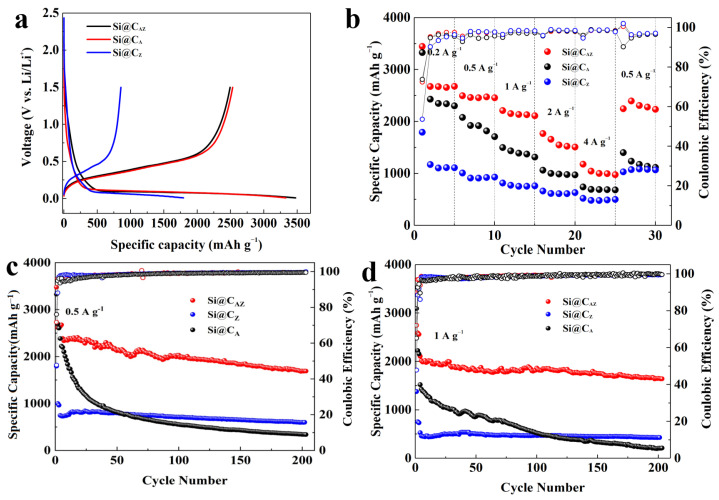
Electrochemical performance of the Si@C_AZ_, Si@C_A_ and Si@C_Z_ anodes: (**a**) first charge/discharge profiles at 0.2 A g^−1^, (**b**) rate capabilities, (**c**) cyclability at 0.5 A g^−1^ and (**d**) cyclability at 1 A g^−1^.

Figure 5a exhibits the first charge/discharge profiles of the Si@C_AZ_, Si@C_A_ and Si@C_Z_ anodes at a current density of 0.2 A g^−1^. Obviously, the initial Li^+^ insertion potential of three anodes is higher than that of the pristine Si anode [37]. Based on the first charge/discharge curve, the Si@C_AZ_ electrode demonstrates a high capacity of 3458.1 mAh g^−1^ and a relatively high ICE of 71.73%. The Si@C_A_ electrode can also deliver a high capacity of 3327.9 mAh g^−1^ with a comparable ICE value. Meanwhile, the Si@C_Z_ only provides a relative low capacity of 1793.1 mAh g^−1^ with a low ICE of 47.7%, which severely hinders the PZS precursor as an individual carbon source to improve the electrochemical performance of Si anode. After deducting the capacity contribution from C_AZ_ (Figure S6), the capacity of Si NPs in Si@C_AZ_ reaches a high capacity of 3567.1 mAh g^−1^, approaching the theoretic capacity of 3579 mAh g^−1^ for Si material [38]. Figure S7 exhibits the charge/discharge profiles of the Si@C_AZ_ anode from the 1st to 30th cycle at 0.2 A g^−1^. The charge curves of 10th, 20th and 30th are almost overlapped, manifesting the good reversibility of the Si@C_AZ_ electrode through partial carbonization strategy. Figure 5b shows the rate performance of the three Si@C electrodes. As can be observed, at current densities of 0.2, 0.5, 1, 2 and 4 A g^−1^, the Si@C_AZ_ electrode can demonstrate high average capacities of 2825, 2469, 2148, 1601 and 1039 mAh g^−1^, respectively. When the current density is turned back to 0.5 A g^−1^, a high capacity of 2292 mAh g^−1^ can still be achieved. In contrast, although the Si@C_A_ has a comparable capacity with that of the Si@C_AZ_ electrode at a low current density of 0.2 A g^−1^, it only can deliver a low capacity of 695 mAh g^−1^ at a high current density of 4 A g^−1^. For Si@C_Z_ electrode, despite the good rate capability, it has low capacity either at the low or high current density. All these suggest that the as-prepared Si@C_AZ_ electrode inherits the advantages of high capacity and high ICE of the Si@C_A_ as well as the good rate capability of the Si@C_Z_ anode.

**Figure 6:**
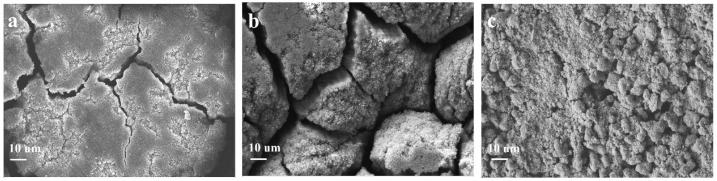
FESEM images of (**a**) Si@C_AZ_, (**b**) Si@C_A_ and (**c**) Si@C_Z_ after cycling.

Cyclability is another indicator for determining the practical performance of an electroactive material. Figure 5c,d shows the long-term cyclability of the three Si@C anodes at 0.5 and 1 A g^−1^, respectively. The Si@C_AZ_ electrode can demonstrate a high charge capacity of 1689 mAh g^−1^ after 200 repeated cycles at 0.5 A g^−1^. Even at a high current density of 1 A g^−1^, after 200 cycles, the Si@C_AZ_ anode still maintains a high capacity of 1643 mAh g^−1^ with a capacity retention of 77.94%. Encouragingly, at the high current density of 1A g^−1^, the capacity loss of the Si@C_AZ_ electrode is only 0.11% per cycle, suggesting outstanding cyclability. By contrast, the capacity of Si@C_A_ electrode continuously fades during the cycling process and only delivers a low capacity of 217 mAh g^−1^ at 1 A g^−1^ after 200 cycles. Although the Si@C_Z_ electrode shows good cyclability, after 200 cycles, it only can deliver low capacity values of 598 and 430 mAh g^−1^ at current densities of 0.5 and 1 A g^−1^ (Figure 5c,d), respectively. The cyclability of the three electrodes proves again that the Si@C_AZ_ electrode inherits the good merits of the two electrodes, i.e., the high initial capacity/high ICE of Si@C_A_ electrode and the good rate capability/cyclability of the Si@C_Z_ electrode. The electrochemical performance of the Si@CAZ electrodes is also very competitive compared to Si@C electrodes prepared by other methods (Table S1). The big difference of the cyclability of the Si@C_A_, Si@C_AZ_ and Si@C_Z_ electrodes can also be verified by their surface morphology after cycling. As shown in Figure 6a, after cycling, the Si@C_AZ_ electrode roughly reserves its pristine flattening surface except for few cracks that formed during the cycling test. Meanwhile, for Si@C_A_ electrode, it broke into small isolated islands and serious crack propagation can be easily observed (Figure 6b). For Si@C_Z_ electrode, although it presents a similar surface feature as that before cycling, a rougher surface and pulverization of some Si@C_Z_ particles can also be observed. In addition, a big cavity resulting from volume contraction of Si@C_Z_ during the delithiation process can also be observed (Figure 6c). The big contrast of the Si@C_AZ_, Si@C_A_ and Si@C_Z_ electrodes after cycling tests is coincident with their electrochemical performance.

**Figure 7:**
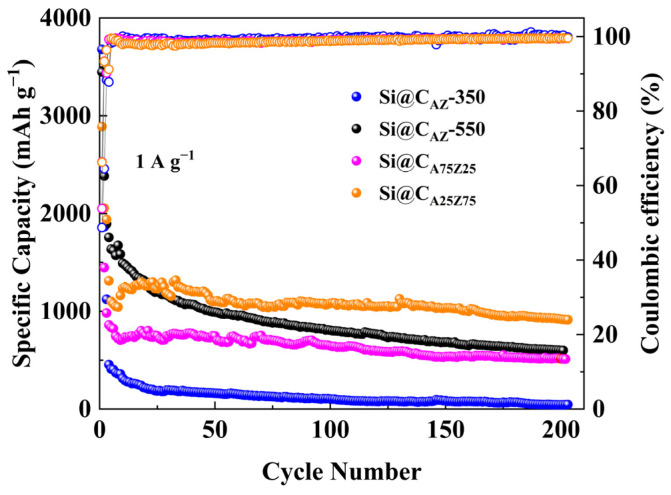
Long-term cycling performance and ICE of Si@C_AZ-_350, Si@C_AZ_-550, Si@C_A25Z75_ and Si@C_A75Z25_ anodes at 1 A g^−1^.

The cyclability of the Si@C_AZ_ electrode at different carbonization temperatures was also evaluated. As shown in Figure 7, the capacity of the Si@C_AZ_ carbonized at 350 ^o^C quickly drops below 500 mAh g^−1^ and reaches nearly zero after 200 cycles due to the insufficient carbonization of PAA–PZS dual polymers in Si@C_AZ_ composite. For the Si@C_AZ_ electrode carbonized at 550 °C, its capacity continuously fades to about ~25% of the initial value after 200 cycles due to the over-carbonization of PAA–PZS dual polymers, which leads to a rimous surface morphology, as observed in Figure S5b. These results further confirm that the optimal carbonization temperature for Si@C_AZ_ electrode is 450 °C. Under this temperature, the effect of the PAA/PZS ratio on the electrochemical performance of Si@C_AZ_ electrode was further investigated. As shown in Figure 7, as the ratio of PAA/PZS was adjusted as 25:75, the Si@C_A25Z75_ electrode shows low capacity but good cyclability and delivers a relatively low capacity of 913 mAh g^−1^ after 200 cycles at 1 A g^−1^. As the ratio of PAA/PZS was adjusted as 75:25, due to the high PAA fraction, the capacity of the Si@C_A75Z25_ electrode rapidly decreases at the initial few cycles and then keeps stable and finally stabilizes at ~550 mAh g^−1^ after 200 cycles at 1 A g^−1^. The big difference in the electrochemical performance of Si@C_AZ_, Si@C_A_, Si@C_Z_, Si@C_A25Z75_ and Si@C_A75Z25_ electrodes suggests that there is a trade-off between the ratio of the PAA to PZS, and only the Si@C_AZ_ electrode with PAA/PZS ratio of 50:50 can deliver optimal electrochemical performance among all electrodes.

The superior electrochemical performance of Si@C_AZ_ electrode can be accounted for by considering the following factors: (1) the polar groups in PAA enable the uniform dispersion of Si NPs and PZS molecules in water and guarantee the uniform coating of C_AZ_ layer on the surface of Si NPs; (2) the residual polar groups in C_AZ_ have a strong interaction with the underlying Cu foil, which, together with the strong interaction between C_AZ_ and Si NPs, form a dual-interfacial bond and thus guarantee the Si@C_AZ_/Cu electrode with strong mechanical strength; (3) the partial carbonized uniform C_AZ_ layer can effectively accommodate the volume expansion of Si NPs and significantly improve the cyclability of the as-prepared Si@C_AZ_ electrode; (4) the partially carbonized conductive C_AZ_ network provides a continuous pathway for the transportation of Li^+^ and electron during the lithiation/delithation process and, thus, avoids the usage of insulating binder and conductive carbon agents in the traditional electrode manufacturing process; and (5) the heteroatoms in C_AZ_ can greatly improve the electrical conductivity of the Si@C_AZ_ composite. More importantly, the synthesis of high-performance Si@C_AZ_ anode is in situ integrated into the electrode manufacturing process and significantly decreases the cost of the LIBs without sacrificing the electrochemical performance of the Si@C_AZ_ anode.

**Figure 8:**
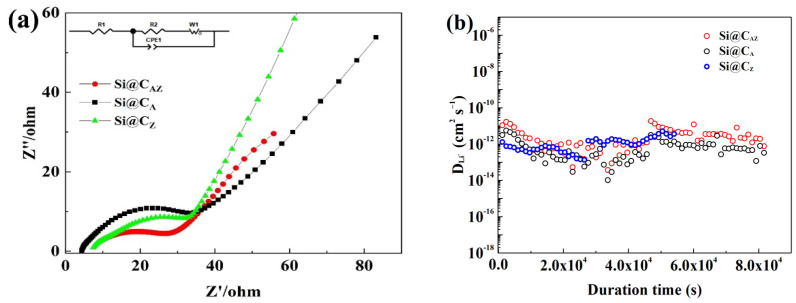
(**a**) Nyquist plots and (**b**) $D_{{Li}^{+}}$ of Si@C_AZ_, Si@C_A_ and Si@C_Z_ electrodes after cycling.

To gain an insight into the performance discrepancy of the Si@C_AZ_, Si@C_A_ and Si@C_Z_ electrodes, the three electrodes were subjected to EIS and GITT measurement. As shown in Figure 8a, the EIS spectra of the three electrodes after cycling are composed of a straight line in the high-frequency region corresponding to the Warburg impedance of Li-ion diffusion into the bulk of active material and a suppressed semicircle in high-/median-frequency region relating to the charge transfer resistance (*R*_ct_) at the electrode/electrolyte interface [39]. The *R*_ct_ value of the three electrodes can be obtained by fitting their EIS spectra based on the equivalent circuit fitting as demonstrated in the inset of Figure 8a. As shown in Table 1, the *R*_ct_ value of the Si@C_AZ_ electrode is only 25.12 Ω, smaller than either that of the Si@C_A_ (32.18 Ω) or that of Si@C_Z_ (35.79 Ω) electrode, implying a lower charge-transfer resistance and faster charge-transfer dynamics at the Si@C_AZ_/electrolyte interface. Figure 8b shows the diffusion coefficients of the Li^+^ ($D_{{Li}^{+}}$) in three electrodes. Although the $D_{{Li}^{+}}$ of the three electrodes is among 10^−11^–10^−13^ cm^2^ s^−1^, the $D_{{Li}^{+}}$ of the Si@C_AZ_ is observably higher than those of the Si@C_A_ and Si@C_Z_ electrodes. The lower *R*_ct_ and higher $D_{{Li}^{+}}$ of Si@C_AZ_ electrode further prove the superior electrochemical performance of the Si@C_AZ_ electrode over those of the Si@C_A_ and Si@C_Z_ electrodes, which is closely related with the partially carbonized conductive C_AZ_ network in providing a continuous pathway for the transportation of Li^+^/electron as well as the heteroatoms (N and P) in increasing the conductivity of the C_AZ_ matrix [40,41].

**Figure 9:**
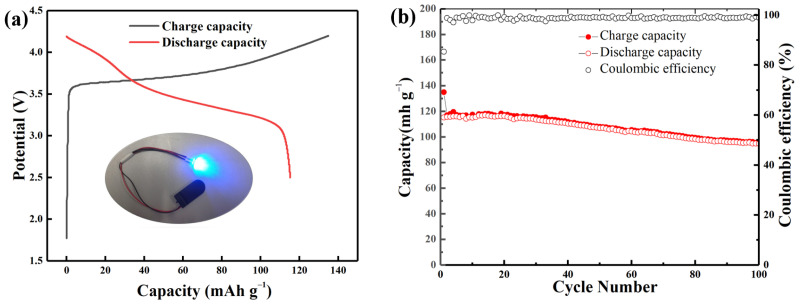
(**a**) The charge/discharge profile and (**b**) cyclability of the assembled Si@C_AZ_||LCO full cell at 0.1 C.

Full cells were assembled to demonstrate the possible applications of the Si@C anode in LIBs with the prelithiated Si@CAZ as anode and commercial lithium cobalt oxide (LCO) as cathode. The assembled Si@CAZ||LCO full cell exhibits charge/discharge capacities of 134.9 and 115.2 mAh g^−1^, respectively, with an ICE value of 85.4% (Figure 9a). The assembled full cell can successfully drive a red light-emitting diode for several hours (inset of Figure 9a). The full cell can also deliver a reversible capacity of 95.3 mAh g^−1^ after 100 cycles with an acceptable capacity retention of 82.7% (Figure 9b). The maximum energy density of the assembled full cell is around 419.3 Wh kg^−1^, with an average working voltage of 3.64 V, which also exceed the current commercial LIBs.

## 3. Materials and Methods

### 3.1. Materials

Polyacrylic acid (Mw = 450,000) was purchased from Sigma-Aldrich (Shanghai, China). 4,4′-sulfonyldiphenol (BPS, Mw = 250.27) was purchased from J&K Scientific Ltd. (Beijing, China). Hexachlorocyclotriphosphazene (HCCP, Mw = 347.66) and methanol were purchased from Aladdin reagent Co., Ltd. (Shanghai, China). Triethylamine (TEA) was purchased from Sinopharm Chemical Reagent Co., Ltd. (Beijing, China). Si NPs with a particle size of 30–60 nm were purchased from Xuzhou Hongwu Nanometer Material Co., Ltd. (Xuzhou, China). All reagents are used without further purification.

### 3.2. Synthesis of PAA–PZS Dual Polymers

The required PZS precursor for preparing Si@C_AZ_ core–shell structure is synthesized through a crosslinking reaction by using HCCP and BPS as raw materials. Typically, 50 mg of HCCP and 113 mg of BPS are dissolved in 10 mL of anhydrous methanol followed by ultrasonication for 5 min. Then, 2 mL of TEA catalyzer is slowly added into the solution to catalyze the reaction. After reacting for 12 h, a white PZS solution is obtained. Finally, 50 mg of PZS solid and 50 mg of PAA are dispersed in 1.5 mL of deionized water under stirring and reacted at room temperature. After reacting for 24 h, the mixed solution of PAA–PZS is freeze-dried and PAA–PZS solid was obtained.

### 3.3. Preparation of Si@C Composites

To prepare Si@C_AZ_, 100 mg of Si NPs and 100 mg of PAA–PZS (PAA:PZS = 50:50, wt%, the same hereinafter) are dispersed in deionized water to form a homogeneous slurry. The resultant slurry was then coated on Cu foil and dried for 12 h in a vacuum oven at 60 °C, followed by heating at 450 °C for 1 h with a ramp rate of 2 °C min^−1^ under Ar atmosphere. After cooling to room temperature, the Si@C_AZ_-coated Cu foil was taken out for structural characterization and electrochemical performance evaluation. The Si@C_AZ_ was also annealed at 350 °C and 550 °C and the synthesized composites were named as Si@C_AZ_-350 and Si@C_AZ_-550, respectively. To simplify the electrode nomenclature, the sample annealed at 450 °C was denoted as Si@C_AZ_ unless otherwise stated. The Si@C_AZ_ electrodes with PAA/PZS ratios (25:75 and 75:25) and annealing at 450 °C were denoted as Si@C_A25Z75_, Si@C_A75Z25_ correspondingly. The individual PAA or PZS was also used as a carbon source to fabricate Si@C composite by using the same procedure and the corresponding Si@C was denoted as Si@C_A_ or Si@C_Z_, respectively.

### 3.4. Material Characterizations

The morphology and microstructure of all prepared samples were observed by using a field emission scanning electron microscope (FESEM, Hitachi-SU8010, North Avenue, Atlanta, GA, USA) and a high-resolution transmission electron microscope (HRTEM, JEM-2100 F, Verdun, France). The functional groups of sample material were characterized by using an MB154S-Fourier transform infrared (FTIR) spectrometer on a pressed KBr pellet with 0.5 wt% ratio of sample. The crystallographic structure of the as-prepared samples was detected on an X-ray Powder diffractometer (XRD-D8 Advance, λ = 1.5406 Å, Bruker, Billerica, MA, USA). The carbon mass fraction in Si@C_AZ_ composite was determined by using a thermogravimetric analyzer (TGA, Netzsch STA 449 F3/F5, Woden, Australia) with a ramp rate of 10 °C min^−1^ within a temperature range of 30–900 °C in air atmosphere.

### 3.5. Electrochemical Measurements

The electrochemical performance of the as-prepared Si@C-based electrodes is evaluated within CR2032 coin-type half-cell configuration. The half-cell was assembled in an Ar-filled glove box by using the Si@C-coated Cu foil as the working electrode, lithium metal as the counter electrode, commercial Celgard 2400 film as the separator, and the 1 M LiPF_6_ dissolved in EC, DEC and DMC (1:1:1, *v*:*v*:*v*) with 10 wt% fluoroethylene carbonate (FEC) additives as the electrolyte. The electrochemical performance of the as-prepared Si@C-based electrodes is evaluated by using a galvanostatic charge/discharge (GCD) technique. The GCD tests were carried out under current densities of 0.2–4 A g^−1^ within a potential window ranging from 0.01 to 1.5 V. Cyclic voltammetry (CV, 0.01–1.50 V) and EIS (frequency ranging from 100 kHz to 0.01 Hz with amplitude of 10 mV) measurements were performed on a commercial electrochemical working station (CHI 660E, Shanghai, China).

## 4. Conclusions

We have successfully prepared a Si@C_AZ_ anode by partially carbonizing the slurry of Si NPs and PAA/PZS dual polymers on Cu foil. Owing to the advantages of core–shell-structured Si@C_AZ_ anode in accommodating the volume expansion of Si NPs and the heteroatom doped C_AZ_ continuous network in promoting the transportation of Li^+^/electron, as well as the strong dual-interfacial bonding between the Si@C_AZ_ and underlying Cu foil, the as-prepared Si@C_AZ_ electrode shows a high specific capacity of 3458.1 mAh g^−1^ at 0.2 A g^−1^ and can still deliver a high capacity of 1039 mAh g^−1^ at a high current density of 4 A g^−1^, suggesting good rate capability. The Si@C_AZ_ electrode also possesses good cyclability and can deliver a high capacity of 1643 mAh g^−1^ with 77.94% of capacity retention at a high current density of 1 A g^−1^ after 200 cycles. More importantly, the good-performance Si@C_AZ_ anode can be simply synthesized by partially carbonizing the slurry of the Si NPs and dual polymers and can be realized in situ during the electrode manufacturing process of lithium-ion batteries, thus significantly decreasing the cost of LIBs but without sacrificing the electrochemical performance of Si@C_AZ_ anode, suggesting its promise in LIBs in the coming future.

## Figures and Tables

**Table 1:** Simulation results of the EIS spectra for three Si@C anodes after cycling.

Sample	R_1_ (Ω)	R_ct_ (Ω)	W_1_-R	W_1_-T	W_1_-P	CPE_1_-T	CPE_2_-P
Si@C_AZ_	5.712	25.12	119.1	0.9946	0.5613	0.000404	0.4645
Si@C_A_	4.232	32.18	225.6	1.423	0.5408	0.000124	0.6861
Si@C_Z_	6.256	35.79	321.9	0.0402	0.3940	0.000600	0.4952

## Data Availability

The data presented in this study are available on request from the corresponding author. The data are not publicly available due to privacy.

## Supplementary Materials

The following supporting information can be downloaded at: https://www.mdpi.com/article/10.3390/molecules29010175/s1. Figure S1. The high-resolution XPS spectra of Si@CAZ anode. Figure S2. The high-resolution XPS spectra of (a) C 1s, (b) O 1s, (c) N 1s and (d) P 2p for Si@C_AZ_. Figure S3. Digital photographs of the as-prepared Si@C_AZ_ and after being folded. Figure S4. TGA curves of Si@C_AZ_. Figure S5. SEM images of (a) Si@C_AZ_-350, (b) Si@C_AZ_-550, (c) Si@C_A25Z75_ and (d) Si@C_A75Z25_. Figure S6 The charge–discharge profiles of the C_AZ_ anode (without adding Si NPs) at 0.2 A g^−1^. Figure S7. Charge–discharge profiles of the Si@C_AZ_ anode at 0.5 A g^−1^. Table S1 Comparison of the synthesis method, carbon source and electrochemical performance of various core–shell-structured Si/C nanocomposites. References [39,42,43,44,45] are cited in the supplementary materials.
