# Supplementary material for: Turning Complexity into Simplicity: In Situ Synthesis of High-Performance Si@C Anode in Battery Manufacturing Process by Partially Carbonizing the Slurry of Si Nanoparticles and Dual Polymers
Source: Molecules. 2023 Dec 28;29(1):175. doi: 10.3390/molecules29010175 (PMC10779834; doi:10.3390/molecules29010175)
Supplement: Supplementary file 1 [file molecules-29-00175-s001.zip › molecules-2727793-supplementary.pdf]

Supplementary Materials

Turning complexity into simplicity: In-situ preparation of high-performance Si@C anode in battery manufacturing process by partially carbonizing of dual polymer/Si slurry

Xiaoxian Liu[a], Juan Liu[b], Xiaoyu Zhao[a], Dianhong Chai[a], Nengwen Ding[a, c], Qian Zhang[a, c], Xiaocheng Li[a, c*]

[a]*Jiangxi Province Key Laboratory of Power Battery and Materials, Faculty of Materials Metallurgy and Chemistry, Jiangxi University of Science and Technology, Ganzhou 341000, P.R. China.*
[b]*Jiangxi Province Key Laboratory of Mining Engineering, School of Resources and environmental Engineering, Jiangxi University of Science and Technology, Ganzhou 341000, PR China.*
[c]*Yichun Lithium New Energy Industry Research Institute, Jiangxi University of Science and Technology, Yichun 360904 P.R. China*

[*] Corresponding authors.
Email: xiaocheng_li@jxust.edu.cn; liujuan@jxust.edu.cn

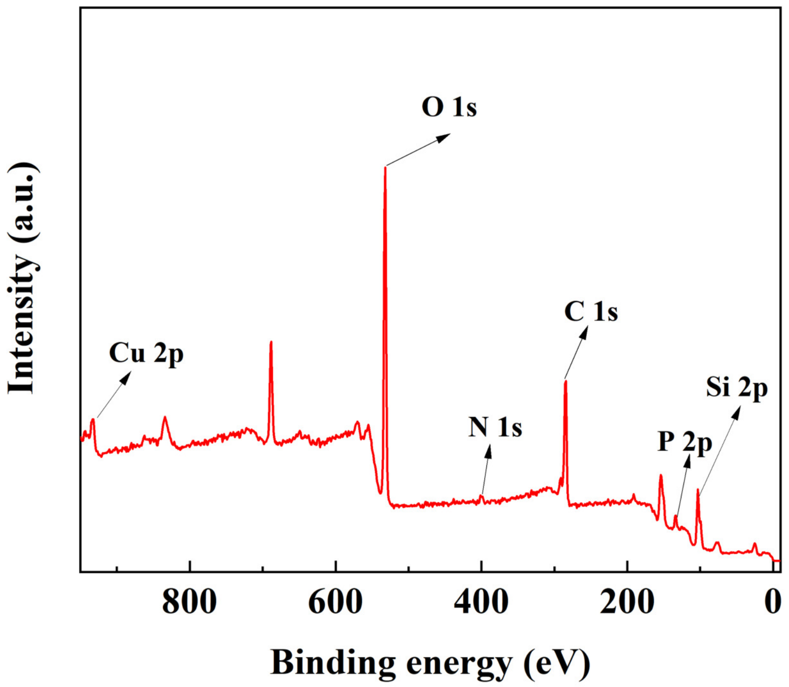

Figure S1. The high-resolution XPS spectra of Si@C_{AZ} anode.

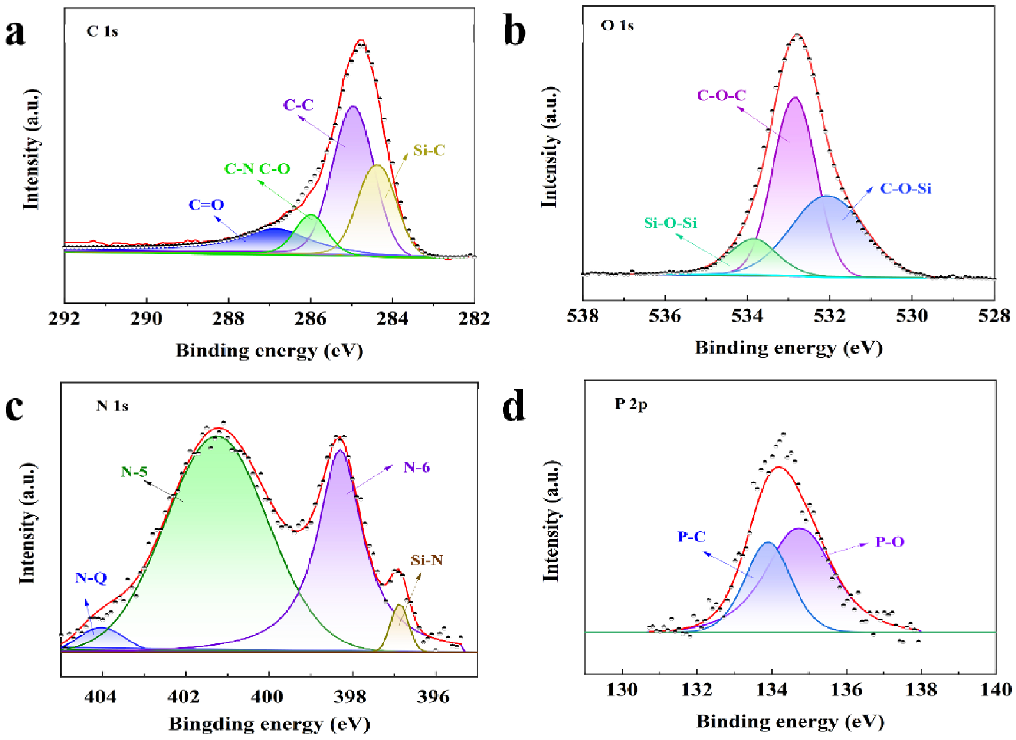

Figure S2 The high-resolution XPS spectra of (a) C 1s, (b) O 1s, (c) N 1s and (d) P 2p for Si@C_{AZ} anode.

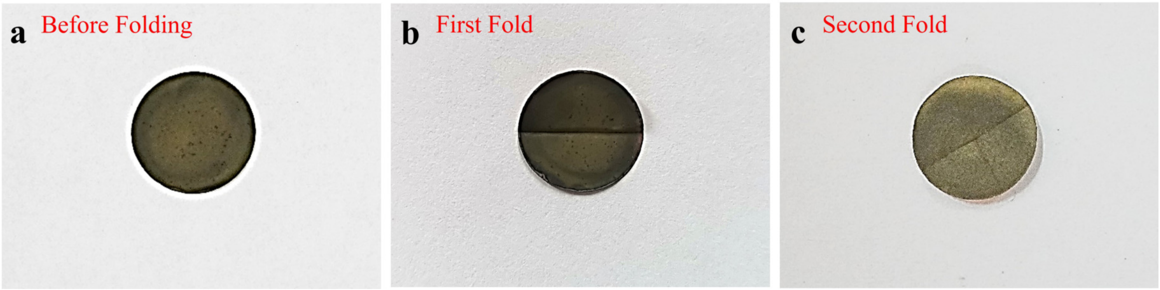

Figure S3 Digital photographs of the as-prepared Si@C_{AZ} and after being folded.

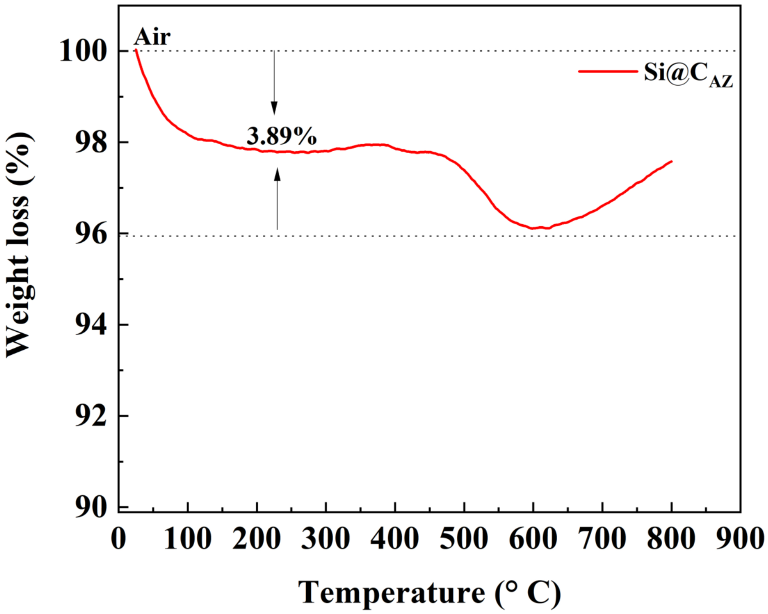

Figure S4 TGA curves of Si@C_{AZ}.

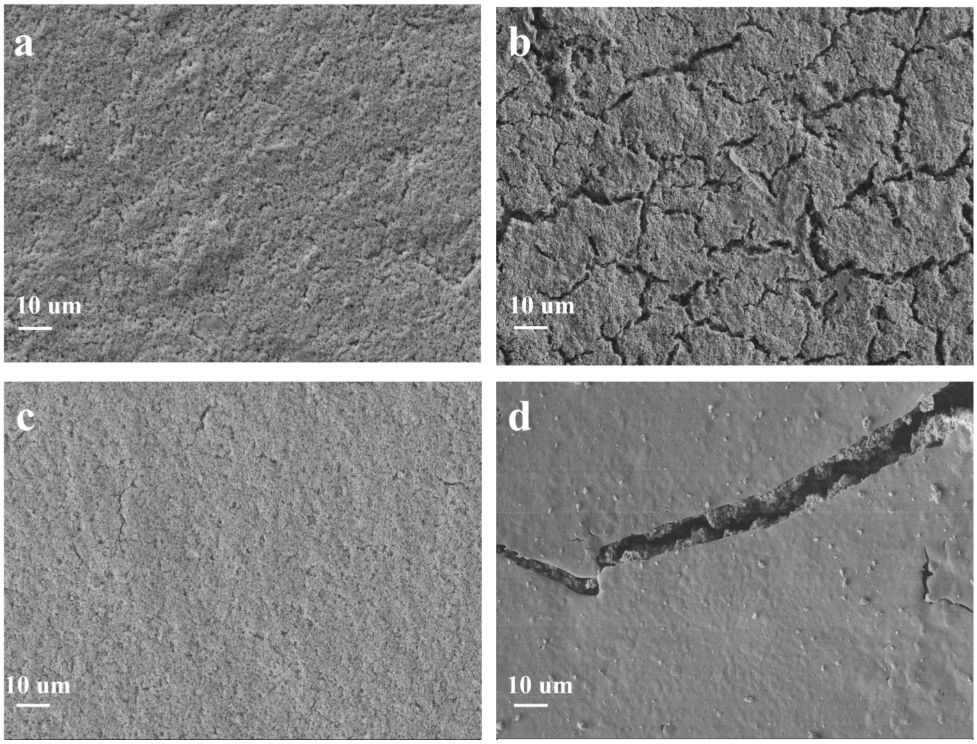

Figure S5 SEM images of (a) Si@C_{AZ}-350, (b) Si@C_{AZ}-550, (c) Si@C_{A25Z75} and (d) Si@C_{A75Z25}.

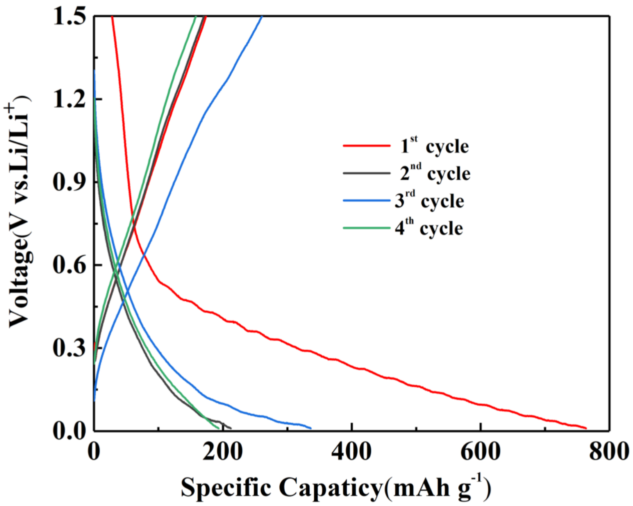

Figure S6 The Charge-discharge profiles of the C_{AZ} anode (without adding Si NPs) at 0.2 A g^{-1}.

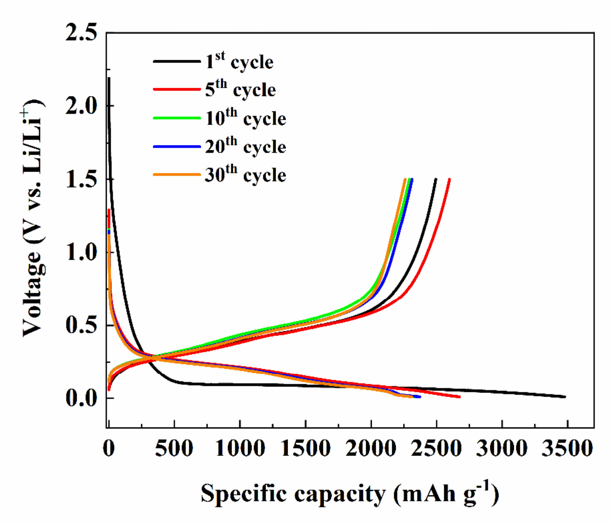

Figure S7 Charge-discharge profiles of the Si@C_{AZ} anode at 0.5 A g^{-1}.

Table S1 Comparison of the synthesis method, carbon source and electrochemical performance of various core-shell structured Si/C nanocomposites.

Sample	Synthesis method	Carbon source	Si (wt%)	Current density (mA g^{-1})	Capacity (mAh g^{-1}) after (n) cycles	Rate capability (mAh g^{-1}@mA g^{-1})	Ref.
Si@10C nanoparticles	Sol-gel	Phenolic resins	81.7	500	1006 (500)	1209 @ 16.8	1
Si@SiOx@C nanocomposites	Hydrothermal	Glucose	75	150	1100 (60)	600 @ 1000	2
Si@C nanoparticles	Pyrolysis	Polyvinylidene fluoride	95.7	50	1290 (30)	450 @ 1000	3
porous Si@C	Vacuum distillation	pitch	75.7	2000	309.5(350)	447 @ 1000	4
Si@TiO2@rGO composite	sol-gel combined with graphene encapsulation processes	graphene	93.43	200	1135.1 (100)	568.1@3000	5
Si@C	partially carbonization	PAA and PZS	96.11	1000	1643 (200)	1039 @ 4000	This work

Reference:

[1] Fang, R., Miao, C., Mou, H., Xiao, W. Facile synthesis of Si@TiO_2@rGO composite with sandwich-like nanostructure as superior performance anodes for lithium ion batteries *J. Alloy. Compd.* **2020**, 818 152884

[2] Hu, Y.S., Demir-Cakan, R., Titirici, M.M., Müller, J.O., Schlögl, R., Antonietti, M., Maier, J. Superior storage performance of a Si@SiOx/C nanocomposite as anode material for lithium-ion batteries *Angewandte Chemie International Edition* **2008**, 47 1645-1649

[3] Luo, W., Wang, Y., Chou, S., Xu, Y., Li, W., Kong, B., Dou, S.X, Liu, H.K.,Yang, J. Critical thickness of phenolic resin-based carbon interfacial layer for improving long cycling

stability of silicon nanoparticle anodes. *Nano Energy* **2016**, 27 255-264

[4] Tao, Y., Tian, Y., An, Y., Wei, C., Li, Y., Zhang, Q., Feng, J. Green and facile fabrication of nanoporous silicon@carbon from commercial alloy with high graphitization degree for high-energy lithium-ion batteries *Sustainable Materials and Technologies.* **2021**, 27 e00238

[5] Xu, Y., Yin, G., Ma, Y., Zuo, P., Cheng, X. Nanosized core/shell silicon@carbon anode material for lithium ion batteries with polyvinylidene fluoride as carbon source. *J Mater. Chem.* **2010**, 20 3216-3620
